# Future Time Perspective Impacts Gain-Related but Not Loss-Related Intertemporal Choice

**DOI:** 10.3389/fpsyg.2018.00523

**Published:** 2018-04-11

**Authors:** Tian Li, Yuxin Tan, Xianmin Gong, Shufei Yin, Fangshu Qiu, Xue Hu

**Affiliations:** ^1^Department of Psychology, Faculty of Education, Hubei University, Wuhan, China; ^2^Department of Psychology, The Chinese University of Hong Kong, Shatin, Hong Kong

**Keywords:** future time perspective, imagine future, intertemporal choice, gain-related choice, loss-related choice, discount rate

## Abstract

Future time perspective (FTP) modulates individuals’ temporal orientation in selecting their motivations and goals, which widely influences their cognitions and behaviors. However, it remains unclear how FTP exactly affects intertemporal choice. To clarify the effect of FTP on intertemporal choice, 90 college students (*M*_age_ = 21.70, *SD* = 1.23) were randomly assigned to the limited FTP condition (16 males, 29 females) and the open-ended FTP condition (17 males, 28 females). In the limited FTP condition, participants were instructed to imagine their states of being 70 years old, whereas in the open-ended FTP condition, they were instructed to describe their current states. All participants then completed a series of intertemporal choice tasks, in which they chose from gain- and loss-related choices occurring at various time points. Results showed that the participants who received the future-imagining manipulation had more limited FTP compared with those who did not receive the manipulation, which confirmed the validity of the FTP manipulation. A 2 (FTP: limited vs. open-ended) × 2 (type of choice: gain vs. loss) repeated measures ANOVA on discount rate revealed a significant interaction between these two factors. The participants in the limited FTP condition had higher discount rates on gain-related choices but showed no difference on loss-related choices compared with the participants under the open-ended FTP condition. The results suggest that limited FTP could lower individuals’ future orientation (i.e., willingness to delay an outcome) on gain-related, but not on loss-related, intertemporal decision-making.

## Introduction

Intertemporal choice involves tradeoffs among costs and benefits occurring at different time points (Frederick et al., 2002). A typical paradigm on intertemporal choice is to ask people to choose between sooner and later gains. People tend to choose the sooner gains, although the later gains are larger in size (Frederick et al., 2002; Green and Myerson, 2004; Berns et al., 2007).

A series of elegant mathematical models have been proposed by economists and psychologists to interpret such a preference for sooner gains (Frederick et al., 2002), such as the exponential discounting model (Samuelson, 1937) and hyperbolic discounting model (Ainslie, 1975). One common idea in these models is that the subjective value or utility of an outcome would be mentally discounted by decision-makers when the outcome is delayed. The degree of discounting can be indexed by a discount rate—a larger discount rate indicates a higher degree of discounting, which means that a sooner gain is more preferred over a later gain (Frederick et al., 2002).

Delay discounting happens not only to gains but also to losses. Discounting of future losses and gains could be described in similar discounting functions (Loewenstein, 1987; Estle et al., 2006). However, losses are usually discounted at lower rates compared with gains, which is termed the sign effect or gain-loss asymmetry (Loewenstein and Prelec, 1992; Frederick et al., 2002; Xu et al., 2009). Loss aversion from the prospect theory (Kahneman and Tversky, 1979) has been applied to interpret such an effect. Loss aversion illustrates that losses have a larger psychological impact compared with gains of the same size, which means that the psychological impact of delayed losses is also larger than delayed gains of the same size (Loewenstein and Prelec, 1992; Frederick et al., 2002). The sign effect also suggests that subjective values of losses are less influenced by delay compared with gains.

As delay discounting involves evaluation and choice of outcomes that will happen in the future, perception of future time is particularly relevant to intertemporal decision-making. Investigations on how perception of speed, length, concomitant cost, and risk of time delay influence intertemporal choice have shown that the temporal discount rate would be higher when a same period of delay is perceived to be slower, longer, more costly, or more risky (Frederick et al., 2002; Löckenhoff et al., 2011).

Future time perspective (FTP), as a critical component of time perception, can also influence delay discounting (Guo et al., 2017). The socioemotional selectivity theory (SST; Carstensen et al., 1999) asserts that individuals’ orientation of life goals is associated with their FTP. In the context of SST, FTP specifically refers to individuals’ subjective perception of the open-endedness of their future time. According to SST, people prioritize future-oriented goals (e.g., acquisition of knowledge) and distal outcomes (e.g., a bright future) more when they perceive their future time as open-ended, whereas people prioritize present-oriented goals and immediate outcomes (e.g., fulfillment of emotional satisfaction) more when they perceive their future time as limited. As people grow older, they perceive future time as increasingly limited, and thus, they gradually change their life goals from future- to present-oriented. The age-related transition in goal orientation resulting from FTP change has been verified, and moreover, it brings widespread and pervasive effects onto cognitions and behaviors, such as attention, memory, social interaction, and decision-making (e.g., Carstensen et al., 1999; Reed and Carstensen, 2012).

According to SST, FTP can affect intertemporal decision-making such that older adults are more present oriented compared with younger adults when making intertemporal choices. Empirical studies have shown that older adults had lower discount rates (i.e., more future oriented) compared with younger adults did (Green et al., 1999; Harrison, 2002; Read and Read, 2004; Reimers et al., 2009; Simon et al., 2010; Jimura et al., 2011; Löckenhoff et al., 2011), which seems to contradict the prediction of SST. One possibility is that age difference in discount rate might be confounded by multiple factors. Indeed, the psychological motives underlying intertemporal choice are complex, including not only perception of time but also factors related to intelligence (Shamosh and Gray, 2007), personality (Wittmann and Paulus, 2008), and sensitivity to rewards (Samanez-Larkin et al., 2011). All these factors could be related to age difference in discount rate. To clarify the effect of FTP on discount rate, the effects of these age-related confounding factors need to be controlled. To achieve the purpose, the present study experimentally manipulated younger adults’ FTP to examine its effect on intertemporal choice.

To control for age-related confounding factors when examining the effect of FTP on intertemporal choice, the present study recruited younger adults only and experimentally manipulated their FTP to see how FTP manipulation alters their discount rate during intertemporal choice. Empirical studies have demonstrated that participants’ FTP could be manipulated by asking them to imagine different scenarios relevant to the open-endedness of future time, such as to imagine a limited or expansive future (Fredrickson and Carstensen, 1990; Fung et al., 1999; Valero et al., 2015). To foreshorten participants’ FTP in the current study, we instructed them to imagine and describe their states of themselves being 70 years old (Ye, 2014).

As limited FTP leads to more focus on present-oriented outcomes, and open-ended FTP leads to more future-oriented outcomes (Carstensen et al., 1999; Freund and Baltes, 2008), we postulated that

Hypothesis 1. Participants with foreshortened FTP (in the limited FTP condition) would have higher temporal discount rates compared with participants who received no FTP manipulation (in the open-ended FTP condition).

As described above, the sign effect, or say, gain-loss asymmetry, in intertemporal choice (e.g., Thaler, 1981; Loewenstein and Prelec, 1992) suggests that loss may be less affected by time perception. We thus expected that

Hypothesis 2. The effect of FTP on temporal discount rate would be smaller for losses than for gains.

## Materials and Methods

### Participants

Participants were 90 college students from Hubei University in China (*M*_age_ = 21.70, *SD* = 1.23). They were randomly assigned to the limited FTP condition (16 males, 29 females, *M*_age_ = 21.49, *SD* = 1.06) and open-ended FTP condition (17 males, 28 females, *M*_age_ = 21.84, *SD* = 1.36). Eight additional participants were excluded, including five who failed in following the instructions and three who did not complete the experiment. The present study was approved by the Ethics Committee of the Faculty of Education in Hubei University in terms of the ethics and safety of psychological experiments. Written informed consent was obtained from all participants. Each participant was paid ¥20 (∼$3.1) at the end of the experiment.

### Materials

#### FTP Scale

The Chinese version of the FTP scale (Fung et al., 2001; Cronbach’s alpha = 0.76) was used to measure subjective perception of future time. The scale consists of 10 items (an example item is “Many opportunities await me in the future”). Participants rated the items on a five-point Likert scale (from 1 = “very untrue” to 5 = “very true”). A higher total score indicates that future time is perceived as more open-ended. The Cronbach’s alpha was 0.72 in the present study.

#### Guidance for Imagination of Future

To make FTP limited, participants were asked to imagine and describe their states of health, cognition, and emotion at the age of 70 years according to the guidance developed by Ye (2014). The guidance includes four open-end questions: (1) “Please imagine and describe your health status when you are 70 years old”; (2) “Please imagine and describe your daily life when you are 70 years old”; (3) “Please imagine and describe your emotional changes when you are 70 years old”; (4) “Please imagine and describe changes in your abilities of cognition, memory, and thinking.” Participants’ answer to each question should consist of 50 words at least. FTP was measured by the Chinese version of the FTP scale after the imagination to check validity of the manipulation.

#### Intertemporal Decision-Making Task

Participants needed to make a series of choices between an immediate gain (or loss) of ¥1000 ($157.7) and a delayed (i.e., 2 months later) gain (or loss) of ¥1050, 1100, 1150, 1250, 1350, 1500, 1700, 1950 ($165.5, 173.4, 181.3, 197.0, 212.8, 236.4, 268.0). The amounts of gain and loss, as well as the length of time interval, were determined according to Tao et al. (2015), which reported that these were sensitive for the detection of experimental effects among young Chinese participants.

### Experimental Design and Procedure

The current study adopted a 2 (FTP: limited vs. open-ended) × 2 (type of choice: gain vs. loss) experimental design, with FTP as a between-subject variable and type of choice as a within-subject variable.

Participants were randomly assigned to either the limited FTP or open-ended FTP condition. Participants in the former condition received FTP manipulation (i.e., imagining their future states), whereas those in the latter condition were asked to describe their current states by four questions similar to the guidance for imagination: (1) “Please describe your current states of health”; (2) “Please describe your daily life”; (3) “Please describe your emotional states”; (4) “Please describe your abilities in cognition, memory, and thinking.” Then, all participants completed the FTP scale. They then turned to 12 gain-related and 12 loss-related intertemporal choice tasks, which were presented on a computer screen by E-Prime 2.0.

In the gain-related intertemporal-choice tasks, the description of the situation reads:

“Suppose that you have participated in a rewarding social activity, and you have two options to get your monetary reward: (1) receive it now; (2) receive it 2 months later. The amounts of money are different in these two options. Please make a choice that you prefer in each of the follow-up pairs of options.”

In the loss-related intertemporal-choice tasks, the description of the situation reads:

“Suppose that you have made a serious mistake in a group activity, and you have to compensate for it by paying money. You have two options to pay: (1) pay it now; (1) pay it 2 months later. The amounts of money are different in these two options. Please make a choice that you prefer in each of the follow-up pairs of options.”

**FIGURE 1:**
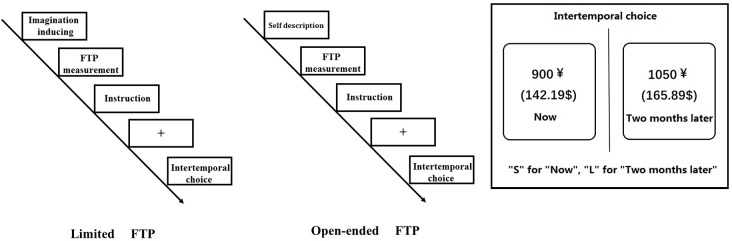
The procedure of the experiment under open-ended vs. limited FTP condition. The choice pairs were presented in random order within the gain/loss condition. The positions (left or right) of the immediate choices and delayed choices were pseudo-randomized, such that the immediate choices were presented on the left side in half of the trials but on the right side in the other half of the trials.

The immediate and delayed options were presented on the left or right side of the computer screen randomly. The order of gain and loss was counterbalanced across subjects (the procedure details are given in **Figure 1**).

### Data Analyses

Participants’ preference for immediate or delayed gain/loss was indexed by the temporal discount rate originated from the hyperbolic discounting function: *V_d_* = *V*/(1+*kd*), where *V_d_* is the subjective value after discounting, *V* is the objective value without discounting, *k* is the discount rate, and *d* is the length of delay (Mazur and Coe, 1987; Frederick et al., 2002; Kazuhisa and Hajime, 2016). To obtain the discount rate (*k*) for each participant, we first identified his/her switching point in the series of intertemporal choice tasks: the point where he/she changed choice from the immediate to a delayed option, or from a delayed to the immediate option. At this switching point, the immediate (representing *V_d_* in the hyperbolic discounting function) and delayed outcomes (representing *V*) had the same subjective value for the certain participants. With these values, the discount rate (*k*) could be calculated for each participant based on the hyperbolic discounting function. All discount rates (*k*s) were then submitted to SPSS 22 for a 2 (FTP: limited vs. open-ended) × 2 (type of choice: gain vs. loss) mixed design, repeated measures ANOVA.

The discount rate is often not normally distributed (e.g., Jones and Rachlin, 2006; Margittai et al., 2015), which violates the assumption of ANOVA. To confirm the reliability of results, we repeated the ANOVA for discount factor *f* (i.e., the immediate value divided by future value at the switching point), which is usually normal distributed.

## Results

### Demographics

Independent *t*-tests showed no significant difference in the participants’ age between the limited FTP and open-ended FTP conditions, *t* = -1.38, *p* = 0.17, Cohen’s *d* = 0.29. No significant difference was found in the level of monthly living consumption between conditions [for the limited FTP condition, *M* = ¥1,153.33 ($181.74), *SD* = ¥209.54 ($33.02); for the open-ended FTP condition, *M* = ¥1,235.56 ($194.70), *SD* = ¥295.54 ($46.57); *t* = -1.52, *p* = 0.13, Cohen’s *d* = 0.32].

### FTP Manipulation Check

The mean score of FTP measured after manipulation was 28.64 (*SD* = 6.51) in the limited FTP condition and 35.11 (*SD* = 4.29) in the open-ended FTP condition. Independent *t*-tests showed that the latter condition had significantly higher FTP scores compared with the former condition (*t* = -5.56, *p* < 0.01, Cohen’s *d* = 1.19), indicating that the limited FTP group had more limited FTP. The results confirmed the validity of the manipulation.

### Analyses of Discount Rate

The discount rates (*k*s) for the different experimental conditions are as follows: for the limited FTP condition, mean *k*_gain_ = 0.15 (*SD* = 0.12) and mean *k*_loss_ = 0.05 (*SD* = 0.09); for the open-ended FTP condition, mean *k*_gain_ = 0.08 (*SD* = 0.07) and mean *k*_loss_ = 0.03 (*SD* = 0.04).

**FIGURE 2:**
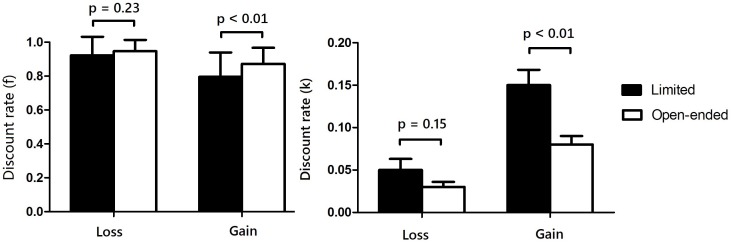
Discount rates of intertemporal choices for gains and losses under limited vs. open-ended FTP condition.

A 2 (FTP: limited vs. open-ended) × 2 (type of choice: gain vs. loss) repeated measures ANOVA on discount rates showed that the main effect of FTP was significant (i.e., higher in the limited FTP condition than in the open-ended FTP condition), *F*_(1,88)_ = 11.68, *p* < 0.01, $\eta_{p}^{2}$ = 0.12; the main effect of types of choice was significant (i.e., higher for gains than for losses), *F*_(1,88)_ = 35.53, *p* < 0.01, $\eta_{p}^{2}$ = 0.30; and the interaction effect was significant, *F*_(1,88)_ = 4.31, *p* = 0.05, $\eta_{p}^{2}$ = 0.05. As shown in **Figure 2**, simple effect analyses showed that the participants with limited FTP had higher discount rates for gains than those with open-ended FTP, *F*_(1,88)_ = 11.39, *p* < 0.01, $\eta_{p}^{2}$ = 0.11; the discount rates for loss between these two FTP conditions had no significant difference, *F*_(1,88)_ = 2.11, *p* = 0.15, $\eta_{p}^{2}$ = 0.02.

To confirm the reliability of results, we repeated the ANOVA for discount factor f (i.e., the immediate value divided by a future value at the switching point), which is usually normal distributed. Discount rates (*f*s) for different experimental conditions are as follows: for the limited FTP condition, mean *f*_gain_ = 0.79 (*SD* = 0.02) and mean *f*_loss_ = 0.92 (*SD* = 0.02); for the open-ended FTP condition, mean *f*_gain_ = 0.87 (*SD* = 0.01) and mean *f*_loss_ = 0.95 (*SD* = 0.01).

A 2 (FTP: limited vs. open-ended) × 2 (type of choice: gain vs. loss) repeated measures ANOVA on discount rates (*f*s) showed similar results with abovementioned: the main effect of FTP was significant, *F*_(1,88)_ = 8.40, *p* < 0.01, $\eta_{p}^{2}$ = 0.09; the main effect of types of choice was significant, *F*_(1,88)_ = 46.75, *p* < 0.01, $\eta_{p}^{2}$ = 0.35; the interaction effect was marginally significant, *F*_(1,88)_ = 3.33, *p* = 0.07, $\eta_{p}^{2}$ = 0.04. As shown in **Figure 2**, simple effect analyses showed that participants with limited FTP had a lower *f* value for gains than those with open-ended FTP, *F*_(1,88)_ = 9.03, *p* < 0.01, $\eta_{p}^{2}$ = 0.10. The discount (*f*s) for loss between these two FTP conditions had no significant difference, *F*_(1,88)_ = 1.47, *p* = 0.23, $\eta_{p}^{2}$ = 0.01.

## Discussion

By manipulating younger participants’ FTP, the current study revealed that FTP modulates discount rates for gains, but not for losses. To be specific, participants under the limited FTP condition discounted gains more than their counterparts under the open-ended FTP condition, but no significant difference was seen in discounting of loss between these two conditions. The results partly confirm our hypotheses that limited FTP would lead to higher discount rates, and that the impact of FTP on discount rate would be higher for gains than for losses.

The results that limited FTP contributes to higher discount rates on gains indicate that perceived open-endedness of future time remarkably affects participants’ intertemporal choice. The finding is consistent with the suggestion of SST (Carstensen et al., 1999) that open-ended FTP makes people focus more on future-oriented motivations, goals, and outcomes. When people have a long period of time ahead to live for, they tend to be more willing to delay gains to achieve more.

Older adults (with limited FTP) have been shown to have lower discount rates than younger adults (with open-ended FTP) (e.g., Harrison, 2002; Jimura et al., 2011), which seems to contradict the speculation derived from SST (Carstensen et al., 1999). We argue that these studies did not well control for other age-related confounding variables, and thus could not be used to infer the effect of FTP on intertemporal choice. The current study manipulated the FTP of younger adults to examine its effect on intertemporal choice, such that the confounding effects of age-related factors were clearly excluded. By doing so, we found that FTP indeed significantly influences participants’ intertemporal choice. Moreover, emerging literature has explained the changes in FTP in different ages from the perspective of “psychological connectedness to the future self” (Urminsky, 2017). People are more likely to be “impatient” and prioritize the present over the future when they perceive a weak link between current and future self, compared with those who perceive a close link between current and future (Hershfield et al., 2011; Urminsky, 2017). In the present study, participants in the imagination group had more limited FTP compared with the control group, and thus, they might perceive a weaker connection between their present and future self so that they preferred instant rewards in decision-making.

We found no significant difference in the discount rates for losses between the limited and open-ended FTP conditions. This result did not verify our hypothesis that limited FTP would increase the discount rates for both gains and losses but supported the hypothesis that FTP impacts discounting of losses less than that of gains. The finding is compatible with the sign effect: people discount losses at lower rates compared with gains in intertemporal choice (Thaler, 1987; Loewenstein and Prelec, 1992). A small increase in loss might bring a psychological impact comparable in terms of size to a psychological impact brought by a larger increase in gain, as asserted by the prospect theory (Kahneman and Tversky, 1979).

It might be arbitrary to conclude that FTP did not impact the discount rate for losses based on our results. Although our settings for the intertemporal decision tasks (i.e., amount of gains/losses and length of delay) were found to be proper in the former studies (Liu et al., 2015; Tao et al., 2015), they may be not sensitive enough to catch the effects of FTP on discount rates for losses. To address this limitation in the current study, future studies may systematically change these settings to verify the impact of FTP on discounting of losses.

Future studies could manipulate older adults’ FTP to examine the effect of FTP on intertemporal choice. Although manipulation of FTP results in similar patterns of cognitive and behavioral changes among younger and older adults (Fredrickson and Carstensen, 1990; Fung et al., 1999; Valero et al., 2015), it is unsure whether this is also the case for intertemporal choice. Future studies could also explore the effect of FTP in different domains. Most studies on intertemporal choice have focused on monetary gains and/or losses, whereas a few studies have revealed that people may discount other items (e.g., food) differently than money (e.g., Frederick et al., 2002). It is thus important to examine the robustness of the FTP effect on intertemporal choice across domains.

## Conclusion

By experimentally manipulating younger participants’ FTP, the current study found that limited FTP led to stronger temporal discounting on gains, but not on losses, compared with open-ended FTP. The finding suggests that FTP is more likely to impact intertemporal decisions on gains than on losses: people are less willing to delay gains when they perceive their future life time is limited. The study provides direct evidence on the relationship between FTP and discount rate in intertemporal choice. This finding contributes to reconciling the contradiction in the literature and supports SST, which asserts a strong relation between FTP and the temporal orientation of motivations and goals.

## Author Contributions

TL, XG, and SY designed the study. FQ participated in the data collection. TL, YT, and XG carried out the statistical analysis and wrote the paper. SY is the principal investigator of this project, and supervised the statistical analysis and the manuscript writing and revision. XH assisted with writing the article. All authors read and approved the final manuscript.

## Conflict of Interest Statement

The authors declare that the research was conducted in the absence of any commercial or financial relationships that could be construed as a potential conflict of interest.
